# Mitotic activity patterns and cytoskeletal changes throughout the progression of diapause developmental program in *Daphnia*

**DOI:** 10.1186/s12860-018-0181-0

**Published:** 2018-12-29

**Authors:** Luxi Chen, Rosemary E. Barnett, Martin Horstmann, Verena Bamberger, Lea Heberle, Nina Krebs, John K. Colbourne, Rocío Gómez, Linda C. Weiss

**Affiliations:** 1 Department of Animal Ecology, Evolution and Biodiversity, Ruhr-University Bochum, NDEF 05/751, Universitaetsstrasse 150, 44780 Bochum, Germany; 2 School of Biosciences, University of Birmingham, Edgbaston, Birmingham, B15 2TT UK; 3 Departamento de Biología. Facultad de Ciencias, Universidad Autónoma de Madrid, 28049 Madrid, Spain

**Keywords:** Dormancy, Diapause, Daphnia, Cytoskeleton, Actin, Tubulin, Embryo development, Temperature, Early embryonic cell cycle

## Abstract

**Background:**

Diapause is a form of dormancy that is genetically predetermined to allow animals to overcome harsh environmental conditions. It is induced by predictive environmental cues bringing cellular activity levels into a state of suspended animation. Entering diapause requires organismal, molecular and cellular adaptation to severely reduced energy flows. Cells must therefore have evolved strategies that prepare them for periods with limited metabolic resources. However, changes that occur on the (sub-)cellular level have not been thoroughly described.

**Results:**

We investigated mitotic activity and we monitored cytoskeletal network changes in successive stages of diapausing and non-diapausing *Daphnia magna* embryos using (immuno-)fluorescent labeling. We find that embryos destined to diapause show a delayed and 2.5x slower mitotic activity in comparison to continuously developing embryos. Development is halted when *D. magna* embryos reach ~ 3500 cells, whereupon mitotic activity is absent and cytoskeletal components are severely reduced, rendering diapause cells compact and condensed.

**Conclusion:**

In the initiation phase of diapause, the slower cell division rate points to prolonged interphase duration, preparing the cells for diapause maintenance. During diapause, cytoskeletal depletion and cellular condensation may be a means to save energy resources. Our data provide insights into the sub-cellular change of diapause in *Daphnia*.

## Background

Many animals are able to withstand environmental challenges by entering a state of innate dormancy, in which metabolic activity comes to a halt. This ‘sit-and-wait-it-out’ strategy requires the correct interpretation of cues indicating environmental degradation, but also provides the predictive competence upon which metabolism can be reinstalled [1, 2]. Dormancy can occur at different developmental stages, depending on how the organism’s life cycle is synchronized to its respective habitat [1, 2]. Diapause is a predictive form of dormancy and is genetically predetermined [3, 4]; it is a widespread adaption to seasonality across different invertebrate taxa, synchronizing life-histories with favourable and resource rich conditions and mitigating the exposure to harsh conditions. Here, the developmental programme is routed away from direct morphogenesis into an alternative diapause programme [3]. In the freshwater crustacean *Daphnia,* diapause is often coupled to sexual reproduction (albeit by some obligate parthenogenetic populations as well [5]). When environmental conditions are favourable, *Daphnia* reproduces via parthenogenesis and the clonal embryos complete their development in the mother’s brood chamber to be released as fully developed juveniles [6–8].

Once the environmental conditions start to deteriorate (e.g. crowding, photoperiod changes, temperature changes, and food limitation [9–14]), *Daphnia* females switch their reproductive strategy so that, genetically identical males are produced first and then haploid oocytes are produced [15]. After sexual mating, these haploid oocytes will be fertilized by haploid spermatozoa, from which diploid embryos will develop under the mechanical protection of a robust coat termed the ephippium. This heavily pigmented structure is formed by the maternal carapace [16]. In the subsequent molting cycle of the *Daphnia* female, the ephippium with the embryos (usually two) is shed and often deposited in the sediment. Sexually produced embryos are thus destined to go into diapause when the embryo reaches gastrula [17]. This strategy allows the increase of genetic variability, and is coupled with the ‘wait-it-out’ approach so that genotypes that meet the environmental requirements hatch to form new parthenogenetic populations [7, 18].

The entrance into this state of suspended animation requires organismal, cellular and molecular alterations at severely reduced energy flows [1, 2, 19, 20]. *Daphnia* can rest in sediments for many years, which means that the sub-cellular organelles and the cytoskeleton matrix must be kept minimalistic, yet with the capability to restart embryo development. The oldest recorded resuscitated *Daphnia* embryo is ~ 700 years old [18], but regularly up to 80 years old *Daphnia* can be hatched [21]. One emerging theme in animal diapause is the involvement of small RNAs, chromatin and histone modifications, insulin and FoxO (forkheadbox protein O3) signalling, and cell cycle arrest [2]. Especially, the cytoskeleton appears to be of central relevance; the embryonic cell permits fast replication of cells and genetic material during mitosis [22, 23]. For accurate and complete mitosis, the cytoskeleton is key, as microtubules form the mitotic spindle, and actin participates (in conjoined action with its associated motor protein myosin), to form the cytokinesis contractile that separates the daughter cells [24]. Furthermore, the cytoskeleton functions as an intra-cellular transport system, which is central to cell function and cell viability [25]. Up to now, such changes on the sub-cellular level, have not been investigated in *Daphnia* species. We here studied embryonic development of sexually and asexually produced embryos of the freshwater crustacean *Daphnia magna.* We compared cell number increase over time in diapause destined embryos to normally developing embryos. Based on our observations, we selected dedicated stages for in situ molecular study before diapause, during diapause, and also upon restart of development. These respective stages were compared with those of non-diapausing embryos. In these stages, we determined mitotic activity and cytoskeletal changes. We were thus able to describe changes in cytoskeletal organization that may explain how energetic constraints associated with hypometabolism are overcome with distinctive cellular adaptations that also permits a fast and efficient re-uptake of cellular activity upon diapause termination.

## Results

We aimed to obtain a deeper insight into the cytological basis of diapause. For that, we first determined cell numbers throughout early embryonic development in asexually and sexually produced embryos. We selected three stages with equivalent cell numbers (stage I and stage II) and dedicated morphological features (stage III) representing equivalent developmental time points. Using these stages, we investigated the cytoskeletal changes in both embryo types.

### Cell number changes during development progression

**Fig. 1:**
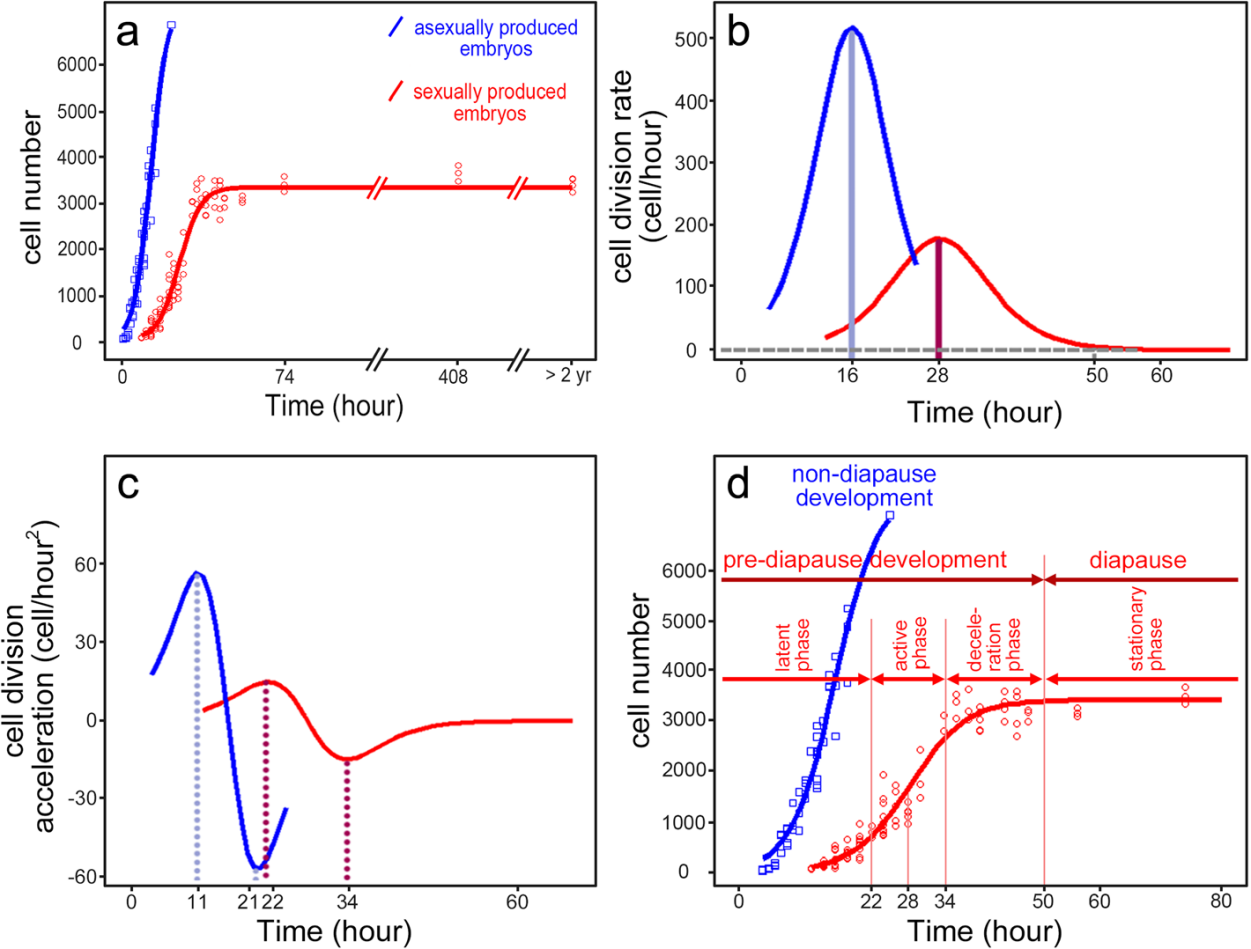
Cellular growth curves based on DAPI stained nuclei in asexually and sexually produced embryos of *Daphnia magna* over time post ovulation. **a** Cell numbers of the asexually produced embryos (blue) increase continuously after ovulation. Cell numbers of sexually-produced embryos (red) are constant at 50 h post ovulation and remain so during the long diapause period (at 4 °C and full darkness). Curves of original data (points) are modelled according to Eq. 1; based on coefficients determined with the logit function in Eqs. 2 and 3. **b** Lines display modelled cell division rate in both embryo types and was calculated as the first derivative of Eq. 1 with respect to time (see Eq. 4). In asexually produced embryos, cell division rate is highest 16 h post ovulation. In sexually produced embryos, the cell division rate is maximal 28 h post ovulation. **c** Lines depict cell division acceleration and was calculated as the second derivative of Eq. 1 with respect to time (see Eq. 5) in both embryo types. In asexually produced embryos acceleration of cell division is maximal at 11 h and minimal 21 h post ovulation. In sexually produced embryos, the maximal acceleration of cell division is observed at 22 h and minimal acceleration at 34 h post ovulation. **d** Descriptive cell growth curves. Based on cell division rate and cell division acceleration embryogenesis can be divided into phases; in asexually produced embryos, there are two phases: i) latent phase: from ovulation to maximum positive acceleration; ii) active phase: the time interval between maximum positive and negative acceleration. These phases are also observed in sexually produced embryos and are then followed by iii) a deceleration phase: the time interval between maximum negative acceleration to the possible cease of cell division (about 50 h post ovulation); iv) stationary phase: from deceleration phase to the end of diapause period

We investigated cell division patterns by counting DAPI stained nuclei during early embryonic development of sexually and asexually produced embryos. Based on the original experimental data, we modeled analytical logistic curves of cell number development over time in asexually and sexually bred embryos (Fig. 1a). During early development of asexually bred embryos, we observed continuous increase in cell numbers. At 25 h post ovulation, we counted over 7000 cells and observed the first morphological traits (i.e. the antennal buds). In sexually produced embryos, the cell numbers showed the classical sigmoidal development curve (Fig. 1a). At 50 h post ovulation, the overall number of cells remained nearly constant with a mean value of 3581 S.D. ±171.5, when not exposed to hatching conditions. Mothers shed the ephippia at around 74 h post ovulation.

Subsequently, we calculated the cell division rate for each time point (Fig. 1 b). In asexually bred embryos, cell division started at 4 h post ovulation and the cell division rate was highest at 16 h after ovulation, where the cell numbers increased by ~ 500 cells per hour. By contrast in sexually produced embryos, cell division started at 12 h post ovulation (at 20 °C) and the cell division rate was highest at about 28 h post ovulation with cell numbers increasing by ~ 200 per hour. At approximately 50 h post ovulation, cell proliferation rate approached zero.

To assess the change of cell division rate, we calculated the cell division acceleration (Fig. 1c). In asexually bred embryos, maximal positive and negative cell division acceleration was observed at 11 h and 21 h post ovulation. By contrast in sexually bred embryos, the maximal positive and negative acceleration was reached at 22 h and 34 h post ovulation, respectively. Based on these time points, we distinguished the cell growth curves into four phases: latent phase, active phase, deceleration phase and stationary phase (Fig. 1d).

Based on the cell number we selected equivalent stages in asexually and sexually produced embryos. Stage I embryos have ~ 1000 of cells, and stage II embryos have ~ 3500 cells. Stage III was based on the appearance of the first visible morphological features i.e. the formation of the second antennae and the abdominal appendages, which occurs by > 7000 cells.

### Mitotic activity

**Fig. 2:**
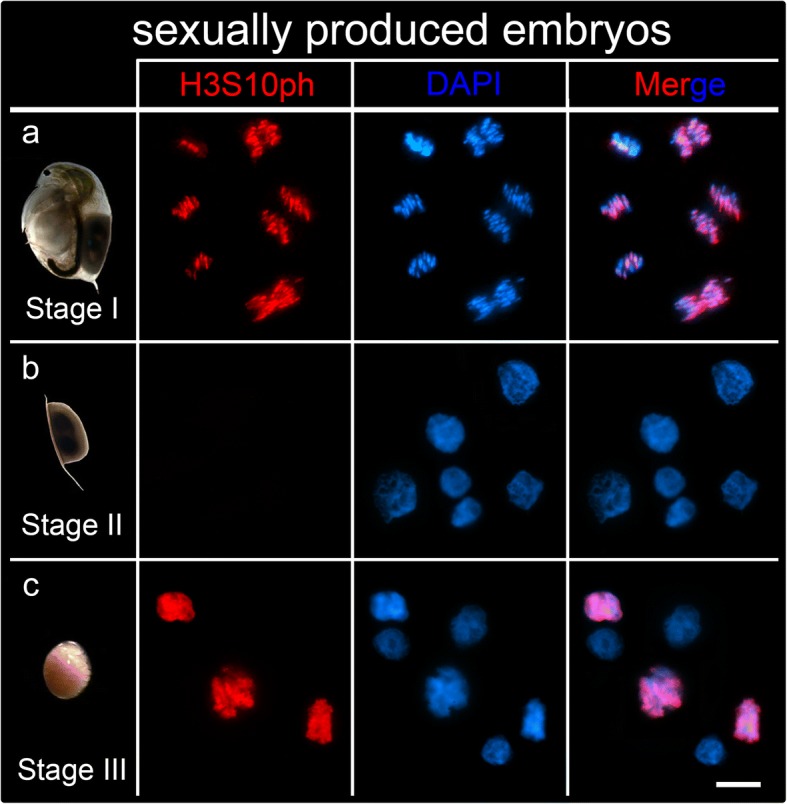
Mitosis in sexually produced embryos. The phosphorylated histone H3S10ph identifies chromatin during metaphase and anaphase (red) which overlaps with chromatin stained with DAPI (blue). **a** Mitotic cells identified with H3S10ph are found in stage I embryos. Metaphases, anaphases and telophases are shown in the image. **b** The HS10ph signal is absent in stage II embryos. These images show several interphases and no dividing cells. **c** Dividing cells showing H3S10ph epigenetic marks are observed in stage III embryos that are resurrected from diapause. Late prophases and metaphases are visible in the image. Scale bars = 10 μm

To precisely determine mitotic activity in sexually produced embryos, we stained the cells with anti-H3S10ph-antibody (commonly known as PH3; Fig. 2). This marker intensely labels the epigenetic modification of phosphorylation of serine 10 of histone 3, which occurs only over condensed chromatin during the metaphase and anaphase stages of mitosis. In stage I, cell division was readily observed (Fig. 2a). In this stage, interphasic cells showing a round nucleus with visible nucleolus were observed to coexist with mitotic cells showing condensed chromatin. In stage II the mitosis marker was completely absent (Fig. 2b), but re-appeared in stage III embryos when diapause is terminated (Fig. 2c).

### Cytoskeletal dynamics

**Fig. 3:**
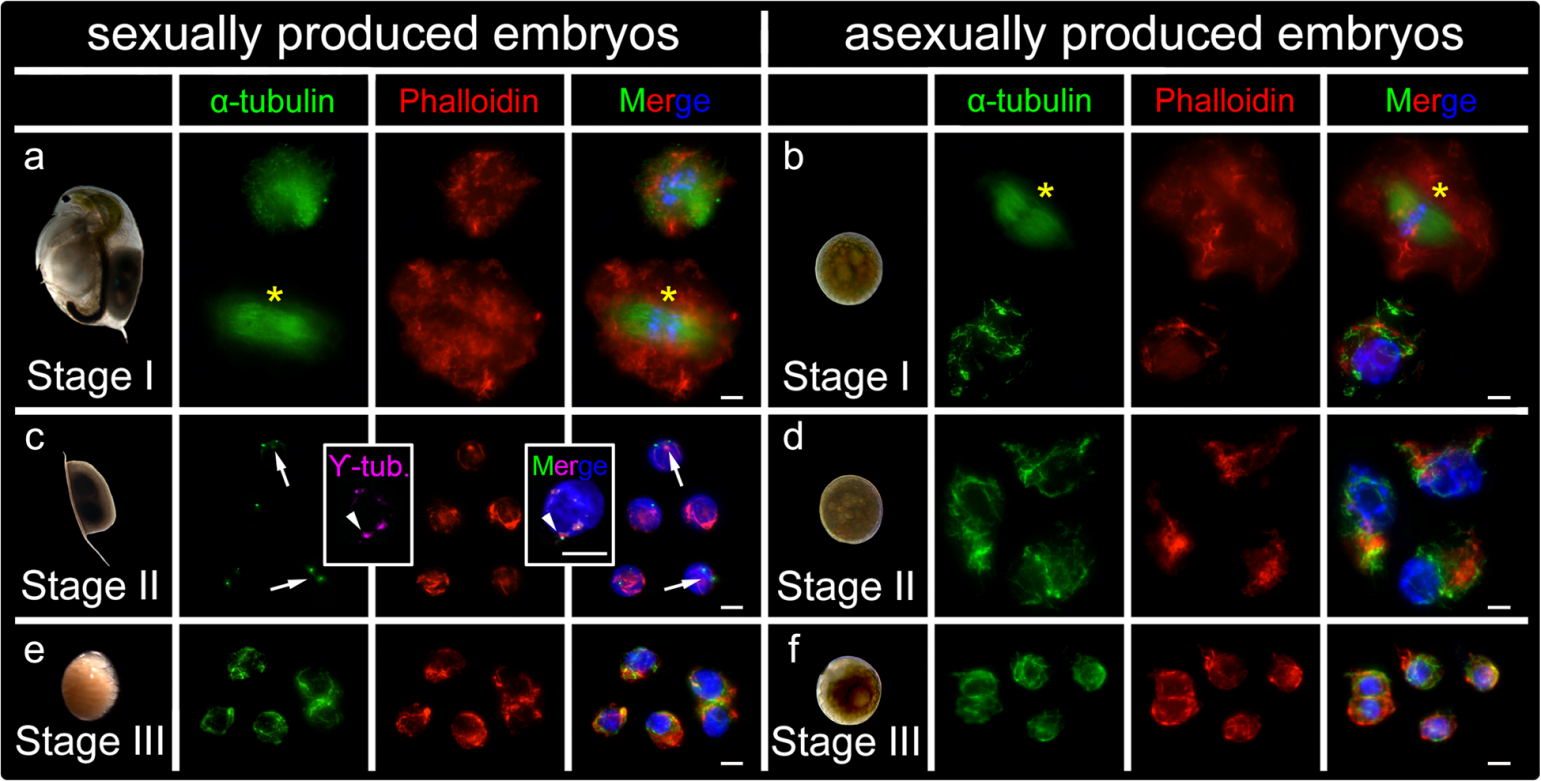
Cytoskeletal changes in cells of sexually and asexually produced embryos of *Daphnia magna*. Double immunolabelling of α-tubulin (green) as marker for microtubules, and Phalloidin (red) as marker for actin filaments, counterstaining of the chromatin with DAPI (blue). **a** α-tubulin and Phalloidin were found in sexually produced embryos and **b** in asexually produced embryos. An interphase and an anaphase (asterisk) are shown in (**a**) whereas an interphase and a metaphase (asterisk) are shown in (**b)**. **c** In cells of diapausing embryos, polymerized microtubules were nearly absent. Only two small green dots (white arrows) are observable; double immunolabelling of α-tubulin (green) is overlaid with pericentriolar material marker γ-tubulin (pink), presumably corresponding with the centrosome localization. Although nucleus size remains constant, actin networks display a significantly reduced cytoplasm during diapause. **d** In asexual embryos no difference between cytoskeletal architecture was observed between stage I and stage II organisms (**e**) in resuscitated *D. magna* sexually produced embryos, cytoskeletal features reappear at stage III accompanied with the onset of cell division. Polymerization of microtubules is visible and actin networks increase in complexity indicating cytoplasmatic growth. **f** Cytoskeleton is continuously expressed in stage III asexually produced embryos. Scale bars: 10 μm

We stained microtubule and microfilament components of the cytoskeleton. For this, we immunolabelled α-tubulin to reveal microtubules, immunolabelled γ-tubulin to detect the pericentrosomal matrix, and Phalloidin to directly reveal actin microfilaments. All the cytoskeleton components studied were detected in both types of stage I embryos displaying a well-developed matrix. In interphasic cells, tubulin produced an intense signal near the nucleus that we propose is the organizing center of microtubules (centrosome). Form the centrosome, microtubules irradiate and expand throughout the wide cytoplasm (Fig. 3a, b; tubulin displayed in green, actin displayed in red). In stage I dividing cells undergoing mitosis, tubulin clearly labelled the mitotic spindle. In all the stages of the cell cycle, actin revealed the expected microfilaments within all the cytoplasm, preferentially near the cell membrane. Remarkably, tubulin detected mitotic spindles, a pivotal apparatus for completing mitosis, in both sexually and asexually produced embryos (anaphase in Fig. 3a, and metaphase in b). In asexually bred embryos, DAPI staining showed classical round-shaped interphase nuclei, with well-formed nucleoli (Fig. 3d). In stage II sexually bred embryos, the tubulin staining was severely reduced to two dots proximal to each other, where double immunolocalization of α-tubulin and γ-tubulin overlaps. No spindle apparati were observed in any of our preparations in sexually bred embryos (Fig. 3c). This suggests that the microtubule network was drastically reduced to the centrosome. Although nucleus size remained similar, actin networks were still detectable but reduced in size and complexity, indicating that the volume of the cytoplasm was also drastically reduced during diapause. Microtubules in stage II asexually bred embryos (Fig. 3d) were similar to stage I embryos displaying widely extended, complex microtubule networks with centrosomes and emanating actin networks (Fig. 3a, b). Interestedly, although the actin networks and cytoplasm of diapause cells were reduced in comparison to asexually produced embryos, nucleus sizes remained constant (Fig. 3c, d). In both stage III embryo types, the cytoskeleton was marked by re-expanding microtubules- and actin-nets of increasing complexity (Fig. 3e, f).

**Fig. 4:**
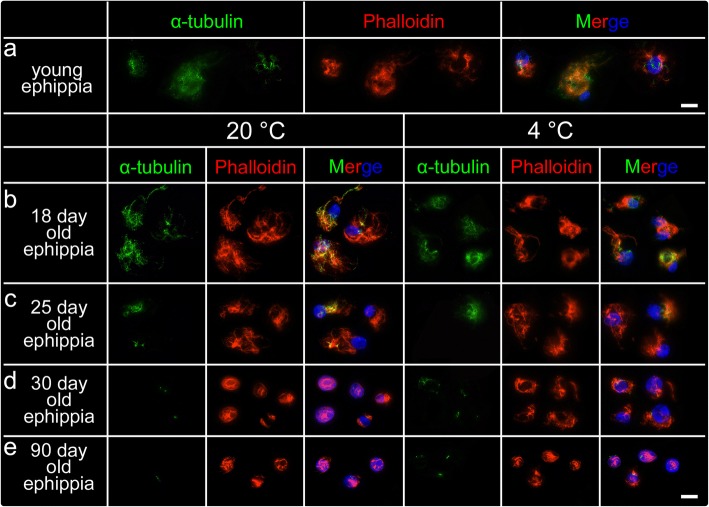
The changes of cytoskeleton in cells of sexually produced embryos of *Daphnia magna* over time at two temperatures. **a** Well-developed cytoskeleton nets were found in cells of young ephippia (about 74 h post ovulation). **b** After 18-day diapause, the cytoskeleton was still detectable, irrespective of temperature conditions at 20 °C and 4 °C. **c** Irrespective of temperature, the microtubule-nets were significantly reduced in 25-day diapausing embryos. Only some microtubular residues were still detectable. Actin networks were expanded. **d** After 30-days of diapause, microtubules were reduced to the centrosome and actin-networks were also reduced in their expansion. **e** After 90-day, all cytoskeletal elements (microtubules and actin microfilaments) were reduced to a minimum. Scale bars: 10 μm

As the stage II sexually produced embryos were kept at 4 °C for ~ 6-month, we wanted to elucidate whether the cytoskeletal reduction was temperature dependent as reported by [26, 27] or whether this is an adaptive feature of cells in diapause. For that, we also traced the cytoskeletal changes across stages in sexually bred embryos that were kept at 20 °C, and 4 °C respectively (Fig. 4). We observed cytoskeletal changes independent from temperature conditions. Again, a well-expanded cytoskeletal network was observed in cells of 18-day-old embryos (Fig. 4a, b). In cells of 25-day-old embryos, the microtubules-nets started dismantling (Fig. 4c). During dormancy progression, the microtubules depolymerized and tubulin pattern of expression was reduced to its core, the centrosome (Fig. 4d, e). In cells of 30-day-old ephippia, microtubule networks were already absent, only showing the centrosome; the actin-nets were clearly reduced (Fig. 4d). In 90-day-old embryos, the cytoskeleton (microtubules and actin microfilaments) were dismantled in concert with a remarkable decrease of the cytoplasm.

## Discussion

During metabolic depression in diapause, cells can sustain long periods of suspended animation, yet maintaining resuscitation capacities and thus viability. In this study, we used sexually and asexually produced embryos of *D. magna* to obtain an insight into the cytological basis of Cladoceran diapause.

### Development in *D. magna* sexually and asexually produced embryos

Cells in asexually bred embryos show typical exponential growth curve within the first 25 h post ovulation. We distinguish four phases: a latent phase, an active phase, a deceleration phase and a stationary phase. While the latent phase may be explained by the low amount of DNA present at this time point in development and therefore difficult to detect, it simply appears to be the initial phase of cell division onset. During the active phase, cells have fast mitotic rates due to the rapid embryonic cell cycle lacking the G_1_ and G_2_ phases [22, 23]. Previous data on classic invertebrate models (e.g. *Drosophila*) suggest that during early embryonic stages, the embryos have large amounts of stored maternal RNA, thereby not requiring G_1_ or G_2_ stages and focusing only on rapidly increasing the number of cells until the mid-blastula transition [23]. During several rapid early embryonic cell cycles in asexually produced embryos, the cell number increases. Deceleration occurs once the cells start to perform G_1_ and G_2_ phases. Thereby cells leave the early embryonic cell cycle and enter the late embryonic cell cycle [23]. Cells begin their own RNA and protein production when tissue differentiation and tissue basal growth begin.

In comparison to asexually produced embryos, in sexually produced embryos the growth curve follows a sigmoidal curve. Differential developmental patterns between sexual and asexual embryos already occur shortly after ovulation. While both embryo types show a latent phase prior to active cell division onset, we observe a delay in the onset of cell division in sexually produced embryos (i.e. the initial increase in cell numbers starts 8 h later than asexual embryos; 4 h vs. 12 h respectively). Similarly, subsequent developmental progression is different in the two embryo types, as we observe differences in the cell division rate during the active phase of embryo development. While sexually bred embryos show a maximal cell division rate of ~ 200 dividing cells per hour (28 h post ovulation), asexually produced embryos show a 2.5x higher cell division rate with ~ 500 dividing cells per hour already 16 h post ovulation. Delayed cell division onset and slower cell division rate may indicate that diapause destined embryonic cells require a longer time to complete the embryonic cell cycle. In this sense, during interphase, cells obtain nutrients which are metabolized, and show highly active nuclei indicating a continuous production of ribosomal subunits to keep up with the high demand of needed proteins (e.g. heat shock proteins were shown to be involved in diapause maintenance [28]). Diapause specific proteins may thus be synthesized prior to diapause entrance. In fact, the protein synthesis rate was shown to be significantly depressed in diapausing annual killifish [28].

The acceleration of cell division reaches its maximum at 11 h post ovulation in asexually bred embryos, then the cell division rate slows at 21 h. By contrast, cell division acceleration is delayed until 22 h in sexually bred embryos and slows at 34 h post ovulation. This indicates that cell numbers stabilize once reaching a maximal cell count coherent with cellular differentiation that we see in asexually produced embryos. In sexual embryos, this event indicates the beginning of the stationary phase and thus developmental arrest and diapause. In fact, from 50 h post ovulation onwards, *D. magna* embryos have a constant cell count of ~ 3500 cells, meaning cell proliferation arrest. This is further validated by the absence of the H3S10ph signal (a marker of condensed chromatin in dividing cells in *D. magna* [29]) in 6 months old diapausing embryos, pointing to a mitotic standstill.

Based on cell counting and cell division modeling, we can identify two essential stages of diapause i.e. initiation and maintenance, according to the classification system introduced by Koštál 2006 [3]. Direct development begins to cease during the initiation phase of diapause, which is marked by a deceleration of cell division rate from 34 h post ovulation to 50 h post ovulation in *D. magna*, when mitotic activity is halted. From 50 h post ovulation onwards, *D. magna* sexually bred embryos reach the maintenance phase of diapause. The maintenance phase of diapause usually occurs in a decisive developmental phase. In *D. pulex* developmental arrest was reported to occur at ~ 1000 cells in early gastrula [30] while brine shrimp arrest development in late gastrula at about 4000 cells [31] so that the precise time point of developmental arrest appears to be species specific, related to the size of the embryos, yet often in a phase of early to late gastrula [32, 33]. Gastrula is an important stage in development, when cell movements separate the embryo into distinct germ layers: endoderm, mesoderm and ectoderm [34, 35]. It is possible that by arresting development in this phase of embryogenesis, this facilitates the scenario for direct resumption of embryogenesis with already predetermined layers of cells with a decided differentiation fate once diapause is terminated.

Diapause is then maintained at a constant cell number until terminated via exogenous stimuli. The physiological processes, if any, during diapause remain vague. However, it is generally accepted that, during this phase, cells encounter metabolic shut down [2, 36]. We therefore investigated the adaptive strategies of cells being exposed to limited metabolic resources, focusing on the cytoskeletal intra-cellular transport system.

### Cytoskeleton

In general, the eukaryotic cytoskeleton of a cell consists of three types of cytosolic fibers: microtubules (polymerized hollow cylinders composed of linear dimers of α/β-tubulin), actin microfilaments and intermediate filaments [37]. In general, the cytoskeleton is responsible for cell motility and maintenance of cell shape, and it plays a crucial role during cell division forming the mitotic spindles and the cytokinesis contractile ring. In addition, the cytoskeleton drives and guides intra-cellular traffic of organelles, ferrying vesicles, molecules and organelles from one part to another part of the cell. For example, the cytoplasmic transport of membrane vesicles is mainly mediated by microtubules [25, 38] assisted by evolutionarily conserved motor proteins, the dyneins [39] and kinesins [40]. As such cargo systems also determine the function of a cell, they are of exceptional importance for cell metabolism and viability. The microfilament actin is known to regulate the cellular assembly, cellular organization and maintains cell-to-cell adhesion. In addition, it gives rise to a cell’s overall size and shape. The intermediate filaments are species-specific and yet to be characterized in *Daphnia* [41, 42]. We therefore monitored cytoskeletal changes throughout the different phases before, during and after diapause.

In stage I of sexually and asexually produced embryos, while cell proliferation was active, the microtubules have fully developed mitotic spindle apparati, indicative of active cell division. A complex network of actin microfilaments determines the shape of the cytoplasm in interphase. This suggests that, also in *Daphnia*, actin microfilaments play an essential role in cell shape and cell migration, both being essential processes of embryogenesis [37]. Actin is also observed in dividing cells in *Daphnia,* where it also participates in shaping the cell and executing the cytokinesis [37, 43]. In stage II diapausing embryos, cells display dematerialization, where the α-tubulin networks are being gradually reduced. Only the two α-tubulin labelled dots, remain in 6 months old diapause embryos. These dots are overlaid with ϒ-tubulin, the evolutionarily conserved component of the pericentriolar matrix that forms a ring from where α/β-tubulin microtubules nucleate and elongate towards the cell membrane [44, 45]. Unlike the microtubules, actin filaments are still observed in the diapause cells, but having a more compact morphology in comparison to cells at other stages. In stage III resuscitated embryos, the cytoskeletal architecture is re-established and cells are morphologically similar to asexually bred embryo cells at the same stage.

Our further experiments confirmed that the cytoskeletal dematerialization can indeed be attributed to diapause. It is not a confounding outcome of low temperature exposure that the embryos experience in the wild and in our experimental conditions [26, 27]. Cytoskeletal dematerialization is not affected by temperature but by time (dematerialization starts at day 25 post ovulation). We observed similar patterns in cytoskeletal structure in both temperatures tested, which suggests that cytoskeletal break-down is likely a reaction to depleted metabolic activity. In fact, our findings suggest that during dormancy, *Daphnia* cells shut down their intra-cellular transportation system. By maintaining the γ-tubulin rings in the centrosome, cells can restore tubulin polymerization and rebuild microtubular networks upon the breaking of diapause. With the maintenance of core centrosomes, the diapausing cells maintain their capacity to efficiently re-start the intra-cellular transport systems once diapause is terminated.

The compact morphology of actin indicates that cells reduce their cytoplasm, which is potentially accompanied by a dehydration of the embryo. Yet, actin can still maintain cell shape and provide stability for the membrane. This also assures intracellular adhesions during diapause and thus the overall morphology of the embryo. It therefore appears that actin is a critical component that can only be slightly reduced, potentially to improve the energy budget [46]. The small cytosolic area may also affect the energy budget enabling short-distance intracellular transport [40, 44]. Resuscitated cells must thus rehydrate, and their cytoplasmic volume increase in concomitance with the growth in length and complexity of the actin and microtubule networks.

While we here only resuscitated 6 months old ephippial embryos, it is commonly known that significantly older diapausing embryos can be hatched [47]. Such adaptive cytoskeletal reorganization has also been reported on the transcriptional level, since a down regulation of actin gene expression in diapause-destined *Artemia* embryos was reported [48].

## Conclusion

We here provide a dedicated and highly resolved staging system of asexually and sexually produced *Daphnia* embryos. With this system, it was possible to determine the time-point of developmental arrest validated by a mitosis marker in diapausing embryos. Furthermore, our data indicate differences in the embryonic cell cycle of diapause destined embryos. To maintain viability and a cost-benefit optimized energy budget, diapausing embryos seem to save energy by disintegrating the tubulin network to save polymerization costs. Meanwhile, a marginally reduced actin network enables continued maintenance of cell shape and inter-cellular cell adhesions, and potentially short-distance intracellular transport.

## Material and methods

### Culture conditions

We cultured a population of different *D. magna* clones (*D. magna* clone Elias from Mount Sinai, Egypt, *D. magna* clone L7 form Lake Ring, Denmark kindly provided by L. Orsini, and *D. magna* clone FT442 from Finland and kindly provided by Dieter Ebert) to study and compare the cellular changes during development in sexually and asexually produced embryos preventing clonal specificities and ambiguities from inbreeding effects [29].

All animals of the culture and the experiments were raised in 1 L glass jars (WECK®, Germany) filled with a modified version of ADaM medium (2.3 mg CaCl_2_ × 2 H_2_0; 2.2 mg NaHCO_3_; 0.1 mg SeO_2_; 12.5 mg sea salt, filled up to 1 L with ultrapure water, refer to Klüttgen, et al. [49]) in temperature controlled incubators at 20 °C ± 0.1 °C and under respective light conditions (for asexually produced embryos: 16:8 day:night; for sexually produced embryos: 8:16 day:night). Animals were fed the algae *Acutodesmus obliquus* ad libitum > 1.5 g C/L. Animal remnants and exuviae were removed every other day and medium was exchanged every week. Unless otherwise stated, all animals were bred in a clone specific manner.

All clonal females were kept in low densities (about 30 adult females per 800 mL of ADaM in a 1 L jar, Weck®; Germany) producing asexual embryos via parthenogenesis. Sexual reproduction in the respective clones was initiated under standardized conditions (20 °C ± 0.1 °C, with a shortened photoperiod 8 h: 16 h light: dark cycle) and via crowding. At densities of more than 50 adult animals and under limited food conditions < 1 g C/L) in 800 mL ADaM this initiated sexual reproduction.

### Ovulation monitoring in sexually and asexually reproducing *Daphnia*

**Fig. 5:**
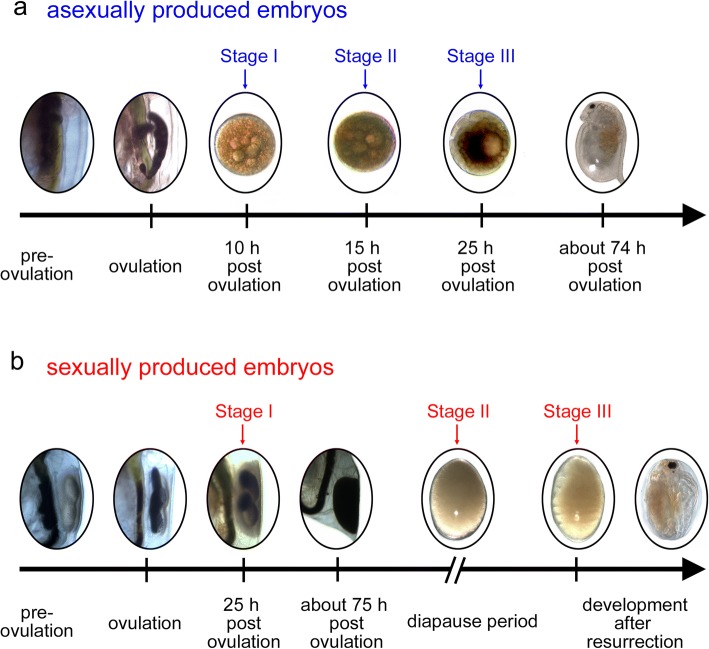
*Daphnia magna* embryogenesis starting with germ cells still in the ovaries. Ovulation and deposition in the brood pouch and maturing ephippium over time in asexually (**a**) and sexually (**b**) produced embryos of *Daphnia magna*. Once (fertilized) germ cells are released from the ovaries, they are deposited in the maternal brood pouch (in asexually bred embryos) or in the developing ephippium (in sexually bred embryos). While asexually bred embryos develop directly, and first signs of morphological features are already observed 25 h post ovulation, sexually bred embryos are maintained in the sclerotizing and darkening ephippium. About 74 h post ovulation the mother molts and thereupon sheds the ephippium. The embryos in the ephippium have arrested in diapause already 50 h post ovulation. Development progresses upon diapause termination by exogenous factors. Stages indicate the time points used for fluorescent (immune)-labelling

To determine how development differs for embryos that are destined to diapause versus those that are not, we monitored the time point of ovulation in sexually and asexually reproducing females. These embryos are distinguishable within the ovary of adult females (Fig. 5), which were individually transferred into 50 mL snap cap vials filled with 40 mL ADaM and algal fed *Acutodesmus obliquus* ad libitum. To ensure fertilisation, we co-cultured one male of a different clone together with one sexually reproducing female. At 15 min intervals, we checked and documented the time point of ovulation. When the animals reached the respective stage for our microscopic observations, they were fixed in 4% PFA-TX (formaldehyde 37%; Merck, Germany; diluted in phosphate buffered saline 0.1 M, pH 7.4; with 0.05% Triton X; Serva, Germany) and stored at 4 °C until further processing. Sexually produced embryos that entered diapause were encapsulated in the maternal ephippia and were cast off during the next molting cycle, at 74 h post ovulation. These ephippia were collected and transferred into dry, cold (4 °C) and fully dark conditions in a refrigerator until processing.

### Cell number changes during developmental progression

In asexually produced embryos, we counted the number of cells hourly from 4 h until 25 h post ovulation. In sexually produced embryos, we counted the number of cells every two hours from 12 h until 40 h post ovulation. We also collected embryos at 44 h, 46 h, 48 h and 56 h after ovulation, before the ephippia were shed. Once the ephippia were cast off, they were collected within 24 h and transferred into dark and cold conditions as described above and collected to be fixed at 408 h post ovulation and 27 months post ovulation.

Fixed embryos were squashed, mounted in Vectashield+DAPI and coverslipped (H-120, Vectalaboratories, Burlington USA) in one single step to ensure that no cells were lost during the preparation procedure. DAPI (4′,6′-diamidino-2-phenylindole) fluorescence staining of the nucleus, by strongly adhering to adenine-thymine rich regions of the nuclear DNA, was documented using a Zeiss Axiophot fluorescent microscope equipped with an Olympus XC10 monochrome digital camera together with the imaging software CellSense (Olympus, Germany).

A single composite image showing all squashed cells of a single embryo was acquired from individual images using the stitching function and cell numbers were determined using the counting function in CellSense (Olympus, Germany). Every time point was collected at least three times.

### Cell division curve modeling

Logistic curves of cell number over time were fitted to the original experimental data with R Studio according to the Eq. (1):

1 $$ y=\frac{a_1}{1+{e}^{-\left({a}_2+{a}_3\times t\right)}} $$

Initial starting parameters were manually set based on the approximated curves.

- y=cell number in one embryo
- a_1_=the first parameter, first a rough approximation of the maximal cell number (e.g. 4000 cells in sexually produced embryos and 8000 cells in asexually produced embryos). Later this value was replaced by the modeled parameter.
- a_2_=the second parameter of the logistic function
- a_3_=the third parameter of the logistic function
- t=hours post ovulation

In detail, a linear model was fitted to the data, using the logit-function to linearize the sigmoid growth curves in Eq. (2).

2 $$ \mathrm{lm}\left(\mathrm{logit}\left(\frac{y}{a_1}\right)\sim t\right) $$

The parameters intercept and slope were extracted from the linear model using the coef()-function and used as approximated values for the parameters a2 and a3.

The final logistic model was established by inserting the calculated rough parameters in the formula and fitting it to the data, using the nls()-function in R, giving the final coefficients for the calculation shown in Eq. (3).

3 $$ {a}_{1/2/3}=\mathrm{coef}\left(\mathrm{lm}\left(\mathrm{logit}\left(\frac{y}{a_1}\right)\sim t\right)\right)\left[1/2/3\right] $$

The logistic model was created by inserting the calculated parameters in the formula. Original data points and the modeled growth curves were plotted in a single plot.

The cell division rate was calculated as the derivative with respect to time to determine the time point of highest cell division rate in Eq. (4).

4 $$ {y}^{\prime }=\frac{\partial y}{\partial t} $$

The second derivative with respect to time was calculated to estimate the change of cell division rate, which is the ‘acceleration’ in cell division using Eq. (5).

5 $$ {y}^{\prime \prime }=\frac{\partial {y}^{\prime }}{\partial t}=\frac{\left(\frac{\partial y}{\partial t}\right)}{\partial t}=\frac{\partial y}{{\partial t}^2} $$

The maxima and minima of this function depicts the transition from the latent phase into the active phase (maxima) and the second transition from the active into the deceleration phase (minima).

### Resuscitation of diapausing embryos

Diapausing embryos used for resuscitation were all 6 months of age (kept at 4 °C in dark conditions). The diapausing embryos were dissected from the collected ephippia and transferred to cell culture dishes filled with 2 mL sterile ADaM medium. Subsequently, embryos were exposed to a constant light source (a combination of Fluora and Biolux lamps; Osram, Germany) at 25 °C. Resuscitation and hatching from the ephippium is normally a process of 3 to 7 days in *Daphnia*.

### Immunolabelling procedure and cytoskeletal staining

For indirect immunofluorescence, we chose three representative stages in both embryo types based on the cell number and embryonic morphology: stage I with ~ 1000 cells (i.e. at 20 °C ± 0.1 °C 10 h post ovulation in asexually produced embryos and 24 h post ovulation in sexually produced embryos); stage II with ~ 3500 cells (i.e. 15 h post ovulation in asexually produced embryos and > 27 months post ovulation dormant embryos); stage III (i.e. diapause termination indicated by the appearance of the appearance morphological features, > 7000 cell stage in asexually and sexually produced embryos). The embryos were fixed for 15 min (in 4% PFA-TX) and squashed on a poly-lysine coated object slide (VWR, Germany). Cover-slips were flipped off with a razor blade upon fixation in liquid nitrogen. All cell preparations were rinsed three times for 5 min in phosphate buffered saline (PBS; pH 7.4 and 0.1 M) and incubated for 2 h with primary antibodies at room temperature. Following three 5 min washes in PBS, the slides were incubated in the dark for 1 h at room temperature with the corresponding secondary antibodies. The slides were then rinsed 3 times in PBS, mounted and coverslipped in Vectashield+DAPI. Finally, coverslips were sealed with rapidly solidifying nail varnish. Preparations were kept in the dark at 4 °C until further analysis.

### Labeling agents

For the immunolabeling procedure, mitotic activity was described with a specific antibody against the cell proliferation marker H3S10ph (06–570; Millipore, Germany) raised in rabbit and diluted to 1:150 in PBS. To detect microtubules, α-tubulin was visualized using a monoclonal FITC (fluorescein isothiocyanate) labelled antibody raised in mouse (F2168; Sigma, Germany) at a dilution of 1:70 in PBS. Specificity of H3S10ph and α-tubulin primary antibody binding has been validated by Gómez et al. [29]. Centrosomal γ-tubulin was detected with the help of a polyclonal antibody raised in rabbit (AB11317; Abcam, Germany) diluted 1:30 in PBS. Primary antibodies were detected with the respective secondary antibodies i.e. goat anti-rabbit IgG (Alexa 594, Dianova Germany), goat anti-mouse IgG (Alexa 488, Dianova Germany), diluted 1:150 in PBS. Fluorescent images were taken as described above.

Polymerized/F-actin microfilaments were directly stained with Phalloidin (Abcam, AB176756; Molecular Probes, Germany) diluted 1:150 in PBS for 2 h at room temperature. Fluorescent images were taken as described above.

### Temperature dependence of cytoskeletal changes

Temperature decline is known to affect cytoskeletal integrity [26, 27]. To ensure that potential cytoskeletal changes are independent of temperature, we exposed resting embryos to 20 °C and 4 °C under dark conditions. Again, the cytoskeleton was stained with Phalloidin and α-tubulin (F2168; Sigma, Germany) as described above. We monitored potential cytoskeletal decay on day 0, day 18, day 25, day 30 and day 90.

### Data analysis and image composition

The morphological changes during development of both embryo types were documented using a stereo microscope (SZX 16 Olympus) equipped with a digital camera (Colorview III, Olympus, Germany) controlled by software Cell^D (Olympus, Germany). Images of one focal plane with adjusted contrast and brightness were assembled using Adobe Photoshop CS6. All data shown in this study were collected from at least six independent replicates.

## Abbreviations

ADaM: Aachender *Daphnia* Medium
DAPI: 4′,6′-diamidino-2-phenylindole
FITC: Fluorescein isothiocyanate
FoxO: Forkhead Box Protein O3
PBS: Phosphate buffered saline 0.1 M, pH 7.4
PFA-TX: Formaldehyde 37% diluted in phosphate buffered saline 0.1 M, pH 7.4; with 0.05% Triton X
PH3/ anti-H3S10ph-antibody: phosphorylation of serine 10 of histone 3
